# Multi-spatial resolution hyperspectral image change detection network integrating feature difference structure

**DOI:** 10.1371/journal.pone.0353888

**Published:** 2026-07-20

**Authors:** Yanhua Xiao, Shuixiang Yu, Wenfeng Li, Huafei Xiao, Yiling Chen, Huayan Zhou, Long Yang

**Affiliations:** 1 School of Information Engineering, Chenzhou Vocation Technical College, Chenzhou, China; 2 Information Center, Hunan Institute of Science and Technology, Yueyang, China; 3 State Key Laboratory of Medical Neurobiology and MOE Frontiers Center for Brain Science, Fudan University, Shanghai, China; Michigan State University, UNITED STATES OF AMERICA

## Abstract

Change detection (CD) can detect changes in the earth’s surface. However, the existing Siamese network-based CD methods usually calculate the Euclidean distance (ED) between two branches to obtain difference features, ignoring the deeper feature disparities captured by the CNN. In addition, the patch obtained in the CD method using spatial information may have the phenomenon that the adjacent pixels and the pixels to be classified are not the same object or change type. To deal with the aforementioned challenges, we propose FDS-BINet, a multi-spatial resolution network integrating a Feature Difference Structure (FDS) and Bicubic Interpolation (BI). The FDS module progressively extracts deep pixel-level differences through residual-enhanced Siamese branches, while BI interpolates the absolute distance spectrum to recover subpixel-level changes. The main contributions of this article are as follows: 1) A change detection framework called FDS-BINet has been proposed that fuses pixel-level and subpixel-level change information. 2) A feature difference structure (FDS) is designed to obtain deep difference features of pixel-level spatial information of bitemporal HSIs; 3) The bicubic interpolation (BI) algorithm is used for absolute distance (AD) spectrum interpolation. We can effectively boost the spatial resolution of AD spectrum data, enabling us to extract feature differences at the subpixel level from bitemporal hyperspectral images (HSIs). The experimental results demonstrate that the proposed FDS-BINet has better performance compared with representative methods in the field of HSI CD.

## 1 Introduction

Hyperspectral images (HSIs) serves as a robust tool for characterizing Earth’s surface properties [1]. The core objective of HSI change detection (CD) is to identify variations by analyzing a pair of images captured over the same geographic coordinates at distinct time intervals [2]. Due to the superior spectral resolution of HSIs, they provide a granular view of land cover attributes, which significantly refines the differentiation of ground objects and enhances the precision of CD algorithms [3].

Typically, the HSI CD pipeline comprises four fundamental stages: preprocessing, feature extraction, difference map classification, and performance assessment [4]. Modern research primarily focuses on advancing feature extraction and classification techniques. Conventional methods often utilized pixel-wise algebraic operations to generate difference images [5]. A seminal advancement was the Change Vector Analysis (CVA) introduced by Malila et al. [6] for forest monitoring. This was later evolved into Improved CVA (ICVA) by Chen et al. [7], which integrated cosine transforms for change categorization and a double-window adaptive step-size approach for threshold determination, thereby bolstering detection reliability.

Furthermore, transformation-based methods aim to isolate change features by projecting HSIs into alternative feature spaces. For instance, Principal Component Analysis (PCA) is frequently employed to mitigate redundant data and lower computational complexity [8]. Nielsen et al. [9] utilized Multivariate Alteration Detection based on canonical correlation analysis, identifying changes by measuring pixel-wise correlations across multitemporal data. Similarly, Slow Feature Analysis (SFA) was introduced by Wu et al. [10] to emphasize transient signals while suppressing stationary features. However, it is important to note that feature mapping processes can inadvertently discard critical information, particularly when faced with high noise levels or suboptimal dimensionality reduction, which may compromise the sensitivity and stability of the detection results. Recent research in related vision tasks has highlighted the effectiveness of dual-teacher feature alignment frameworks [11] in maintaining robustness under adverse conditions, suggesting that cross-modal or cross-temporal feature consistency is vital for reliable intelligent processing.

In the realm of HSI CD, image classification-based approaches primarily bifurcate into joint classification frameworks [12] and post-classification comparison schemes [13]. The latter determines change status by independently categorizing pixels in multitemporal images and subsequently contrasting their class labels. Recent advancements have introduced sophisticated modules to this pipeline: Sun et al. [14] developed a Semantic Structure-aware Inference technique to generate high-fidelity class attention maps without extra training overhead; Wu et al. [15] utilized a Graph-Segmenter to capture intricate local pixel relationships and refine object boundaries; and a Soft-GNN framework was proposed by Wu et al. [16] to mitigate label noise through dynamic data selection. Additionally, specialized architectures like BS3-LNet [17] for anomaly-weighted reconstruction, MSDFFN [18] for multiscale feature refinement, and HyGS-TAN [19] for spectral similarity filtering have pushed the boundaries of detection accuracy. Furthermore, Eigen-CNN [20] has demonstrated the efficacy of eigendecomposition in denoising. Despite these strides, classification-based methods often suffer from cumulative errors and tend to overlook the inherent correlations between image pairs.

Classic deep learning frameworks [21] were widely used in HSI CD methods, and deep learning-based methods [22] have emerged in the HSI CD field. Zheng et al. [23] proposed a CNN-based multi-scale cross-attention network (MSCANet) to extract image information at different spatial resolutions, and they introduced cross-attention modules to boost the model’s ability to understand semantic-level ch-anges between dual-temporal images. Liu et al. [24] proposed an Attention-based Multi-scale Transformer Network (AMTNet) that can effectively mo-del contextual information in dual-temporal images. To tackle the problem of sample imbalance, Han et al. [25] proposed the Hierarchical Attention Network (HANet), which can integrate multi – scale features and refine detailed features. Gong et al. [26] proposed a novel network with spectral and spatial attention that can adapt spectral and spatial information. Lin et al. [27] established connections between image pairs through linear outer product operations and learnt feature representations of bitemporal HSIs through a new Siamese structure. Wang et al. [28] proposed a CD framework with spectral-spatial-wise attention based on adaptive spectral and bitemporal HSI spatial weights for CD contributions. Self-supervised learning has also been applied to change CD in HSIs. To reduce the interference of inherent Gaussian noise in HSIs to CD, Ou et al. [29] proposed a framework with self-supervised contrastive learning pre-training model. To address the label noise issue in unsupervised domain adaptation, Li et al. [30] proposed the Robust Self-training with Label Refinement (RSLR) method, which provides reliable pseudo-labels for target samples. Wang et al. [31] applied residual networks to HSI CD, which was able to build spatial and spectral dependencies adaptively. Considering that the resolution of bitemporal HSIs obtained from different platforms is not necessarily consistent, Chen et al. [32] proposed a fully convolutional change detection framework with generative adversarial networks to address the issue that unsupervised change detection models rely on traditional pre-detection methods. However, the aforementioned methods did not consider subpixel information existing between HSI pixels to supplement the amount of information for CD.

Nevertheless, a critical gap remains: current models frequently neglect the subpixel information residing between discrete HSI pixels. While pixels appear contiguous macroscopically, microscopic subpixel variations provide vital supplementary cues. Conventional patch-based spatial operations often risk mixing heterogeneous objects, necessitating the integration of multiple spatial resolutions to synthesize subpixel details. Furthermore, many Siamese architectures rely on simplistic Euclidean Distance (ED) calculations for difference extraction [28], which fails to fully exploit the deep latent features captured by convolutional neural networks (CNNs).

To address the aforementioned constraints, we propose a novel framework that synergistically leverages bitemporal HSI data at both pixel and subpixel granularities. To capture raw pixel-level spatial characteristics, a Siamese structural design is employed to independently extract deep features from the dual temporal phases. Concurrently, to exploit subpixel-level details, we introduce a specialized interpolation technique applied to the Absolute Distance (AD) spectrum. Specifically, rather than performing interpolation on the original images—which might introduce artifacts or distort change-related information—our approach first computes the AD spectrum between bitemporal image pairs to isolate initial change cues. This AD spectrum is subsequently interpolated to enhance the density of the change information. The primary contributions of the proposed FDS-BINet are summarized as follows.

1. A HSI CD framework with multiple spatial resolutions is proposed to combine pixel-level and subpixel-level change information.
2. A feature difference structure (FDS) is designed to obtain deep difference features of pixel-level spatial information of bitemporal HSIs.
3. The bicubic interpolation (BI) algorithm [33] is used for AD spectrum interpolation, which effectively improves the spatial resolution of AD spectrum and extracts subpixel-level feature difference of bitemporal HSIs.

## 2 Methods

This section delineates the technical architecture and methodological components of the proposed FDS-BINet. We commence with a comprehensive architectural overview of the framework to establish the overall data flow. Subsequently, we provide a rigorous exposition of the core modules, including the bicubic interpolation algorithm for subpixel refinement, and the dual-branch weighting mechanisms (channel-wise and spatial-wise). We also detail the feature extraction process designed to capture discriminative bitemporal representations. The section concludes with an analytical discussion of the loss function designed to supervise the network’s optimization.

### 2.1 Overview of FDS-BINet

**Fig 1:**
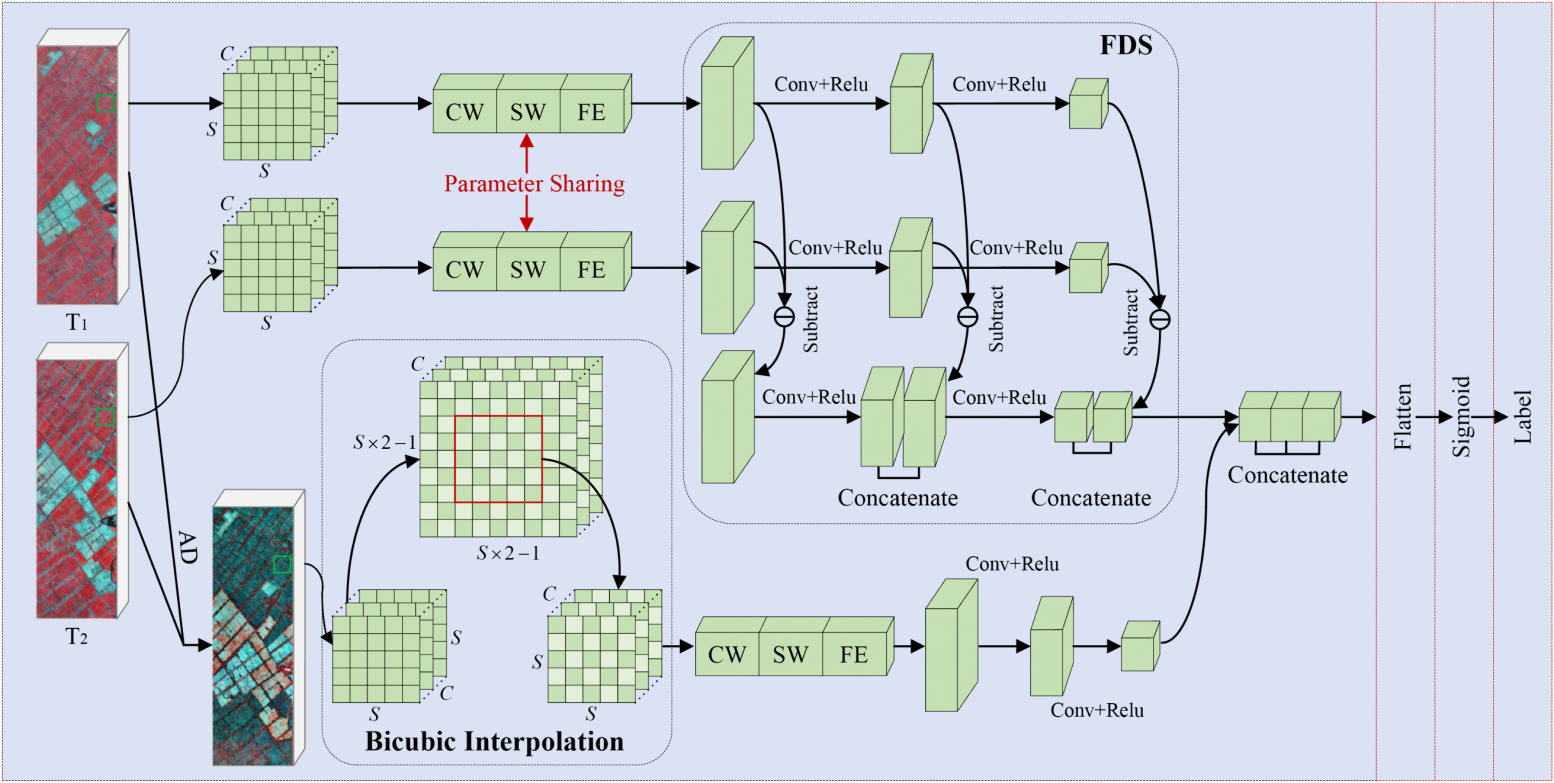
Overview diagram of FDS-BINet.

The comprehensive architecture of the proposed FDS-BINet is illustrated in Fig 1. Our framework maximizes the utility of bitemporal HSIs by integrating information across both pixel and subpixel scales. To capture pixel-level spatial attributes, a Siamese network configuration is employed. Initially, two spatial patches of dimension S×S×C are extracted from identical coordinates across the different temporal phases. These patches undergo refinement through the Channel Weighting (CW) and Spatial Weighting (SW) modules to reconstruct pertinent spectral-spatial information. Subsequently, the Feature Extraction (FE) module derives deep representations from the reconstructed patches, while the Feature Difference Suppression (FDS) component isolates profound differential features between the two Siamese branches. Simultaneously, to harness subpixel-level details, we first calculate the Absolute Distance (AD) between the dual-temporal images. Spatial patches of size S×S×C are extracted from this AD spectrum and processed using the Bicubic Interpolation (BI) algorithm to uncover fine-grained inter-pixel variations. A localized patch of size S×S×C, centered on the target pixel and containing enriched subpixel information, is then isolated. The subpixel-level feature is first processed by two Conv-BN-ReLU blocks for spatial alignment, and the concatenated multiresolution feature is further refined by additional convolutional layers before classification. Specifically, each convolutional layer adopts a (3×3) kernel, stride 1, valid padding, and 32 output channels. After the pixel-level and subpixel-level features are concatenated along the channel dimension, the fused feature is flattened and fed into the final classifier for binary change detection.

### 2.2 Bicubic interpolation algorithm

The BI algorithm is used here to complement the subpixel information of the AD spectrum. The core of the BI algorithm is to calculate the weights of the nearest 16 pixels, and then compute their weighted sum to be the pixel value at the interpolation position. The bicubic basis function for computing 16 pixel weights is defined as


$$\begin{array}{c} W(s)=\left\{ \begin{array}{c} (a+2){\left|s\right|}^{3}-(a+3){\left|s\right|}^{2}+1\;,\left|s\right|\leq 1\\ a{\left|s\right|}^{3}-5a{\left|s\right|}^{2}+8a\left|s\right|-4a\;\;,1<\left|s\right|<2\\ 0\;,otherwise \end{array}\right.\;, \end{array}$$ (1)

where *a* is −0.05 in this paper, *s* is the distance, and *W*(*s*) is the weight converted from the distance between pixels.

**Fig 2:**
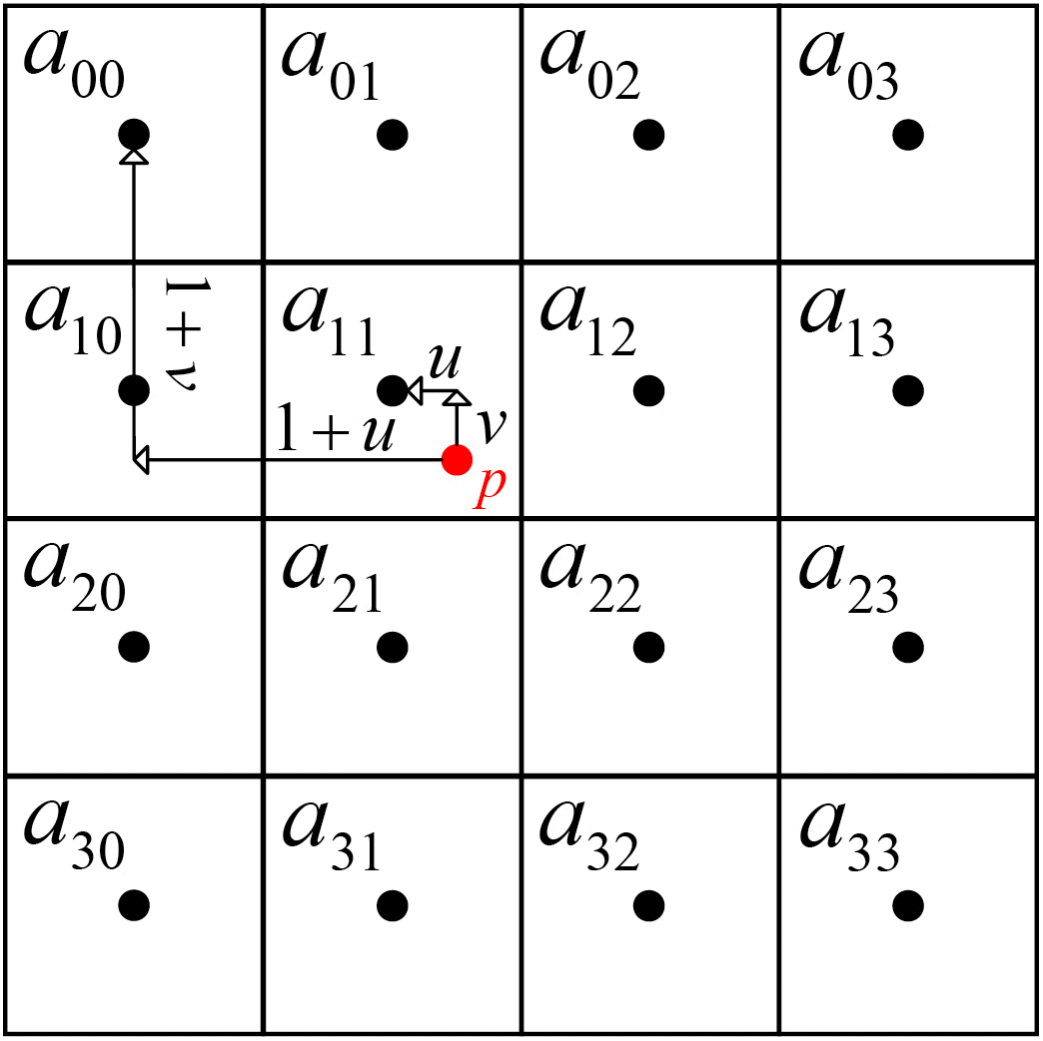
Schematic diagram of the distance between the *p* and *a*_00_.

Fig 2 shows the calculation method of the distance between the interpolation position and the adjacent pixels, where *p* represents the pixel to be interpolated. The bicubic function is one-dimensional, while the pixel is two-dimensional, so we calculate the row and column distances separately. In Fig 2, we define the abscissa distance and ordinate distance between *p* and *a*_11_ as *u* and *v*, respectively. Obviously, the values of *u* and *v* are both in the range of 0–1. So the abscissa distance and ordinate distance between *p* and *a*_00_ can be expressed as 1 + *u* and 1 + *v* respectively. Let the abscissa weight be *h*(0) = *W*(1 + *u*), and the ordinate weight be *o*(0) = *W*(1 + *v*). Similarly, the remaining abscissa weights are *h*(1) = *W*(*u*), h(2)=W(1−u), and h(3)=W(2−u). The remaining ordinates are weighted as *o*(1) = *W*(*v*), o(2)=W(1−v), and o(3)=W(2−v). From the above derivation process, pixel value (*p*) is calculated:


$$\begin{array}{c} p=\sum_{i=0}^{3}\sum_{j=0}^{3}a_{ij}\times h(i)\times o(j)\; \end{array}$$ (2)

where $a_{ij}$ represents the pixel value of the corresponding coordinate position in Fig 2.

The Bicubic Interpolation part in Fig 1 is just a schematic diagram. In the actual implementation process, we perform bicubic interpolation on the entire AD spectrum, and then take out the spatial patch centered on the pixel to be classified.

### 2.3 Channel and spatial enhanced feature extraction module

#### 2.3.1 Channel weighting module.

**Fig 3:**
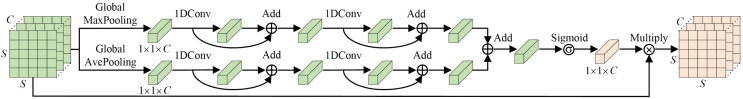
Illustration of CW.

Band information is reconstructed in the CW module and assigned weights adaptively. Fig 3 shows the structural details of the CW module. Given an input spatial patch $P\in {\mathbb{R}}^{S\times S\times C}$, global max-pooling and average-pooling are applied to generate feature vectors $C_{\text{max}}\in {\mathbb{R}}^{1\times 1\times C}$ and $C_{\text{ave}}\in {\mathbb{R}}^{1\times 1\times C}$. For each pooled feature *C*_pool_ (representing *C*_max_ or *C*_ave_), two residual-connected 1D convolutions are performed:


$$\begin{array}{c} C_{pool}^{add_{1}}=\text{Conv1D}(C_{\text{pool}})+C_{\text{pool}} \end{array}$$ (3)

$$\begin{array}{c} C_{pool}^{add_{2}}=\text{Conv1D}(C_{pool}^{add_{1}})+C_{pool}^{add_{1}} \end{array}$$ (4)

The residual features from both branches are merged, and channel weights are generated via a Sigmoid function:


$$\begin{array}{c} C_{\text{weights}}=\text{Sigmoid}(C_{\text{max}}^{add_{2}}+C_{\text{ave}}^{add_{2}}). \end{array}$$ (5)

Finally, the weights are applied to the input patch to reconstruct features:


$$\begin{array}{c} P_{\text{cw}}=P⊗C_{\text{weights}}. \end{array}$$ (6)

#### 2.3.2 Spatial weighting module.

**Fig 4:**
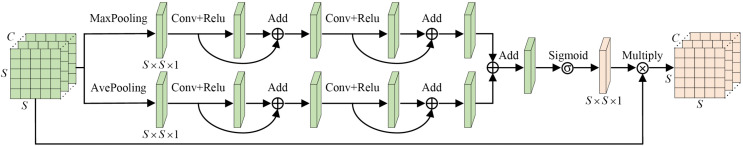
Illustration of SW.

Spatial information is reconstructed in the SW module, so that the pixels that are beneficial to the classification task have greater weight. Fig 4 shows the structural details of the SW module. Given an input patch $P\in {\mathbb{R}}^{S\times S\times C}$, max-pooling and average-pooling generate single-channel maps $M_{\text{max}}\in {\mathbb{R}}^{S\times S\times 1}$ and $M_{\text{ave}}\in {\mathbb{R}}^{S\times S\times 1}$. For each pooled map *M*_pool_ (representing *M*_max_ or *M*_ave_), two residual-connected 2D convolutions with ReLU activation are performed:


$$\begin{array}{c} M_{pool}^{add_{1}}=\text{ReLU}(\text{Conv2D}(M_{\text{pool}}))+M_{\text{pool}} \end{array}$$ (7)

$$\begin{array}{c} M_{pool}^{add_{2}}=\text{ReLU}(\text{Conv2D}(M_{pool}^{add_{1}}))+M_{pool}^{add_{1}} \end{array}$$ (8)

The merged features from both branches generate spatial weights via Sigmoid:


$$\begin{array}{c} M_{weights}=Sigmoid(M_{max}^{add_{2}}+M_{ave}^{add_{2}}). \end{array}$$ (9)

The output is reconstructed as:


$$\begin{array}{c} P_{\text{sw}}=P⊗M_{\text{weights}}. \end{array}$$ (10)

This module focuses on salient regions through hierarchical residual learning and adaptive spatial attention.

#### 2.3.3 Feature extraction module.

**Fig 5:**
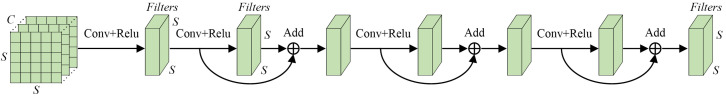
Illustration of FE.

The FE module is used to extract the image features of the reconstructed spatial patch, and its structural details are shown in Fig 5. Given input $P\in {\mathbb{R}}^{S\times S\times C}$, initial convolution extracts primary features:


$$\begin{array}{c} F^{c_{1}}=\text{ReLU}(\text{Conv2D}(P)). \end{array}$$ (11)

Three residual stages sequentially refine features through:


$$\begin{array}{c} F^{c_{k}}=\text{ReLU}(\text{Conv2D}(F^{c_{k-1}}))+F^{c_{k-1}} \end{array}$$ (12)

where k∈{2,3,4}. $F^{{\text{c}}_{4}}$ is the final output of FE. Max-Pooling and Average-Pooling are jointly used because they provide complementary information. Max-Pooling emphasizes the most prominent responses in spectral channels or spatial positions, which helps capture salient change-related cues. In contrast, Average-Pooling describes the overall feature distribution and provides more stable contextual information. Therefore, their combination enables the CW and SW modules to generate more robust channel-wise and spatial-wise weights.

### 2.4 Loss function

HSI CD is a binary classification task. After obtaining the pixel-level and subpixel-level difference features, we need a suitable loss function to optimize the model. In this paper, the binary classification cross-entropy loss function is used as the loss function of FDS-BI, and its equation is:


$$\begin{array}{c} L=-\frac{1}{N}\sum_{i=1}^{N}\left(y_{i}\log{\hat{y}}_{i}+(1-y_{i})\log(1-{\hat{y}}_{i})\right)\;, \end{array}$$ (13)

where *N* is the total number of samples, $y_{i}$ is the marked changed and unchanged label, and ${\hat{y}}_{i}$ represents the predicted probability value calculated by the softmax function.

## 3 Experiments

In this part, we conduct comprehensive experiments to test the experimental performance of FDS-BINet. First, three HSI CD datasets are introduced. Second, we reveal the details of the experimental setup, such as evaluation metrics, comparison methods, and experimental hardware information, etc. Third, all experimental tables and CD maps are displayed and analyzed. Fourth, we further analyze the ablation experimental results in detail. Fifth, the parameters influence in the model is obtained based on the experimental results.

### 3.1 Experimental HSI CD datasets

To validate the efficacy of FDS-BINet, we employed three widely recognized benchmark datasets: Farmland [34], River [35], and Hermiston [36].

**Fig 6:**
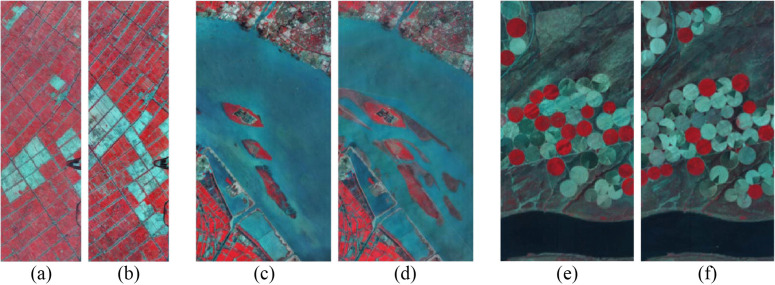
Pseudo-color images of the three experimental datasets.

1) *Farmland Dataset:* As illustrated in Fig 6(a) and 6(b), this dataset covers a spatial extent of 450×140 pixels. After removing noise-prone channels, 155 spectral bands were retained for experimental analysis [34]. The pseudo-color visualizations are synthesized using bands 46, 25, and 18. The predominant change detection target in this scene is the transition of vegetation coverage within agricultural plots.
2) *River dataset:* The River dataset, depicted in Fig 6(c) and 6(d), encompasses a 463×241 spatial area with 198 spectral bands designated for detection tasks [35]. We utilize bands 46, 25, and 18 for pseudo-color rendering. The change dynamics in this dataset primarily involve morphological shifts in the river course and the evolution of adjacent suburban or urban structures.
3) *Hermiston Dataset:* Fig 6(e) and 6(f) display the Hermiston dataset, which features a spatial resolution of 390×200 and 224 reserved spectral bands [36]. Pseudo-color maps are generated from bands 56, 25, and 18. This dataset focuses on the environmental variations occurring between riparian zones and terrestrial land covers.

Following the protocol established in [35], the training samples are balanced with an unchanged-to-changed pixel ratio of 2:1. Furthermore, we adopt a sparse labeling strategy where only 5% of the total pixels are utilized for training, leaving the remaining 95% for performance evaluation. Detailed sample distributions for these three datasets are summarized in Table 1.

**Table 1:** Ratio of training and test samples for three datasets.

	Training			Test		
	Unchanged	Changed	Ratio	Unchanged	Changed	Ratio
Farmland	2100	1050	5%	42623	17227	95%
River	3720	1860	5%	98165	7838	95%
Hermiston	2600	1300	5%	65414	8686	95%

### 3.2 Experimental setup

To quantitatively demonstrate the superiority of FDS-BINet, we adopt a comprehensive evaluation framework comprising Overall Accuracy (OA), Kappa coefficient (Kappa), Precision (Pr), Recall (Re), and F1-score (F1). These metrics are derived from the confusion matrix elements, specifically: True Positives (TP), False Positives (FP), True Negatives (TN), and False Negatives (FN). Following the established formulations in [37], the metrics within our evaluation system are defined as follows:


$$\begin{array}{c} OA=\frac{TP+TN}{TP+FN+FP+TN}\;, \end{array}$$ (14)

$$\begin{array}{c} Kappa=\frac{OA-Pc}{1-Pc}\;, \end{array}$$ (15)

$$\begin{array}{c} Pr=\frac{TP}{TP+FP}\;, \end{array}$$ (16)

$$\begin{array}{c} Re=\frac{TP}{TP+FN}\;, \end{array}$$ (17)

$$\begin{array}{c} F_{1}=2\times \frac{Pr\times Re}{Pr+Re}\;, \end{array}$$ (18)

$$\begin{array}{c} Pc=\frac{\left(TP+FP)(TP+FN)+(TN+FN)(TN+FP\right)}{{\left(TP+FN+FP+TN\right)}^{2}}\;. \end{array}$$ (19)

The metrics OA, Kappa, and F1 serve as indicators of the comprehensive effectiveness of the change detection task, where higher values signify superior performance. Specifically, Pr (Precision) represents the proportion of correctly identified positive instances relative to all predicted positives; a lower Pr suggests a higher FP (False Positive) rate, implying that more unchanged pixels were erroneously classified as changed. Conversely, Re (Recall) denotes the fraction of actual positive samples that were correctly identified; a reduction in this value indicates a diminished capacity to detect genuine changes.

In our implementation, the spatial patch size is set to (9×9×C), where C denotes the number of spectral bands. The AD spectrum is enlarged by bicubic interpolation with an interpolation rate of 2, and a (9×9×C) patch is extracted from the interpolated AD spectrum as the input of the subpixel-level branch. The number of convolutional filters is set to 32 for all datasets.

To rigorously evaluate the proposed FDS-BINet, we benchmark it against nine representative CD methodologies: the classical CVA and PCA-KM, the deep learning-based 2D-CNN, and more advanced frameworks including BCNNs [27], SSA-SiamNet [28], CDFormer [38], CWB-MSSANet [39], SFBS-FFGNET [4], and DIEFEN [40]. In addition, we design three ablation experiments to test the effectiveness of FDS and sub-pixel spatial information. Remove the FDS and subpixel-level branches, and replace the FDS with the traditional calculation of the distance between the upper and lower branches to convert the difference features, named Siam-DisNet. Only the subpixel-level branch of FDS-BINet is removed, named Siam-FDSNet. Additionally, the model with only the FDS module removed is named Siam-BINet. In terms of experimental settings, all comparison methods that need to be trained uniformly should in accordance with Table 1.

### 3.3 Experimental results

**Fig 7:**
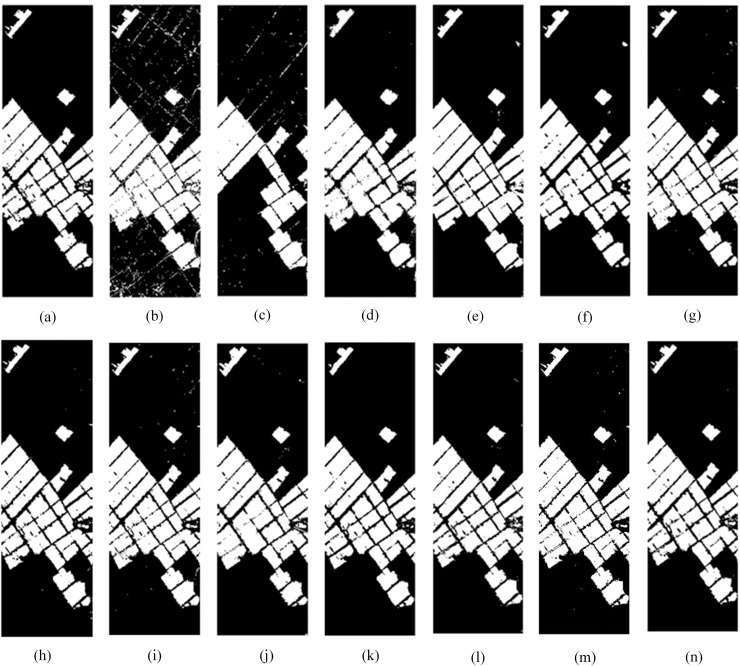
CD binary maps on the Farmland dataset: (a) ground truth; (b) CVA; (c) PCA-KM; (d) 2D-CNN; (e) BCNNs; (f) SSA-SiamNet; (g)CDFormer; (h) CWB-MSSANet; (i) SFBF-FFGNET; (j) Siam-DisNet; (k) Siam-BINet; (l) Siam-FDSNet; (m) DIEFEN; (n) FDS-BINet.

**Fig 8:**
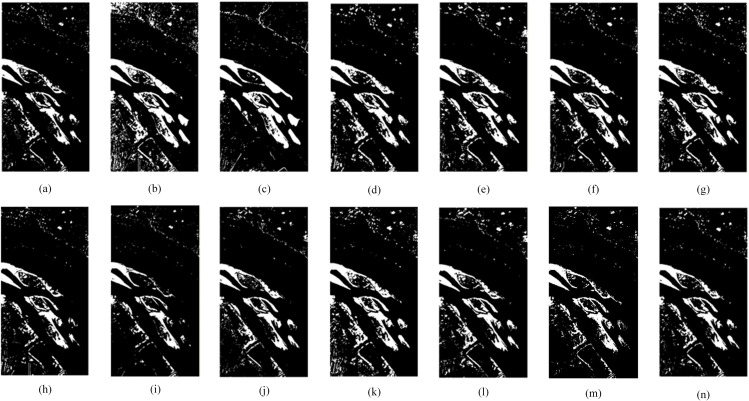
CD binary maps on the River dataset: (a) ground truth; (b) CVA; (c) PCA-KM; (d) 2D-CNN; (e) BCNNs; (f) SSA-SiamNet; (g) CDFormer; (h) CWB-MSSANet; (i) SFBF-FFGNET; (j) Siam-DisNet; (k) Siam-BINet; (l) Siam-FDSNet; (m) DIEFEN; (n) FDS-BINet.

**Fig 9:**
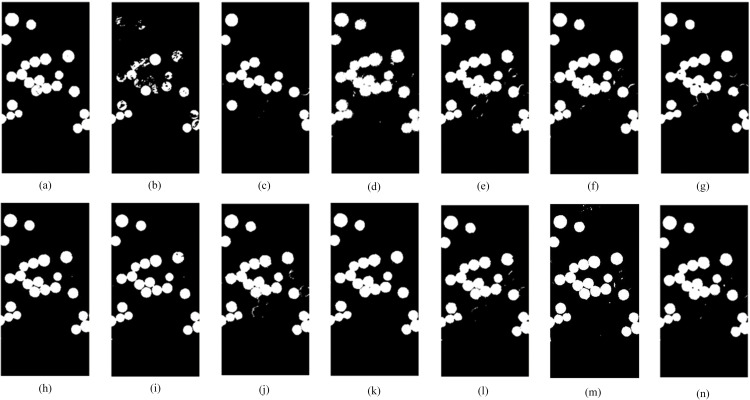
CD binary maps on the Hermiston dataset: (a) ground truth; (b) CVA; (c) PCA-KM; (d) 2D-CNN; (e) BCNNs; (f) SSA-SiamNet; (g)CDFormer;(h) CWB-MSSANet; (i) SFBF-FFGNET;(j) Siam-DisNet;(k) Siam-BINet; (l) Siam-FDSNet; (m) DIEFEN; (n) FDS-BINet.

To ensure a rigorous and unbiased assessment, all evaluated methodologies—with the exceptions of the unsupervised CVA and PCA-KM—were implemented under identical experimental configurations as previously delineated. The comprehensive quantitative findings are documented in Tables 2–4, while the corresponding binary change maps are visualized in Figs 7–9. A detailed, independent performance analysis for each benchmark dataset is presented below:

**Table 2:** Experimental data on the Farmland dataset.

Algorithm	OA	Kappa	Pr	Re	F1
CVA	0.9296	0.8393	0.8108	0.9877	0.8906
PCA-KM	0.8676	0.6502	0.8741	0.6350	0.7356
2D-CNN	0.9603	0.9047	0.9097	0.9572	0.9329
BCNNs	0.9654	0.9170	0.9171	0.9673	0.9415
SSA-SiamNet	0.9738	0.9370	0.9363	0.9754	0.9555
CDFormer	0.9687	0.9237	0.9553	0.9361	0.9456
CWB-MSSANet	0.9647	0.9386	0.9610	0.9513	0.9654
SFBS-FFGNET	0.9807	0.9553	**0.9877**	0.9574	0.9689
DIEFEN	0.9811	0.9546	0.9488	**0.9881**	0.9680
FDS-BINet	**0.9831**	**0.9588**	0.9702	0.9711	**0.9706**

**Table 3:** Experimental data on the River dataset.

Algorithm	OA	Kappa	Pr	Re	F1
CVA	0.9267	0.6575	0.5444	**0.9627**	0.6955
PCA-KM	0.9212	0.5620	0.5363	0.6939	0.6050
2D-CNN	0.9571	0.7336	0.6514	0.9019	0.7564
BCNNs	0.9470	0.6847	0.5947	0.8889	0.7126
SSA-SiamNet	0.9667	0.7819	0.7189	0.9014	0.7999
CDFormer	0.9730	0.8271	0.8578	0.8266	0.8419
CWB-MSSANet	0.9665	**0.9530**	**0.9489**	0.9556	0.9324
SFBS-FFGNET	0.9612	0.8801	0.8107	0.8793	0.8329
DIEFEN	0.9646	0.7711	0.8105	0.7672	0.7904
FDS-BINet	**0.9774**	0.8446	0.8048	0.9161	**0.8568**

**Table 4:** Experimental data on the Hermiston dataset.

Algorithm	OA	Kappa	Pr	Re	F1
CVA	0.9392	0.6608	**0.9872**	0.5317	0.6912
PCA-KM	0.9521	0.7505	0.9671	0.6481	0.7761
2D-CNN	0.9754	0.8901	0.8340	0.9869	0.9040
BCNNs	0.9863	0.9366	0.9006	**0.9927**	0.9444
SSA-SiamNet	0.9846	0.9288	0.8938	0.9860	0.9376
CDFormer	0.9855	0.9336	0.9649	0.9199	0.9419
CWB-MSSANet	0.9834	0.9231	0.9214	0.9473	0.9663
SFBS-FFGNET	0.9693	0.9487	0.9524	0.9403	0.9342
DIEFEN	0.9868	0.9416	0.9327	0.9664	0.9492
FDS-BINet	**0.9895**	**0.9510**	0.9252	0.9909	**0.9569**

1) Quantitative and Qualitative Analysis on the Farmland Dataset: As detailed in Table 2, the unsupervised algebraic approaches, CVA and PCA-KM, exhibit a significant performance gap compared to deep learning-based methods. Among the baseline models, SSA-SiamNet demonstrates a clear advantage over classical architectures like 2D-CNN and BCNNs, achieving a superior OA (0.9738) and F1-score (0.9555). While the performance of 2D-CNN, BCNNs, CDFormer, and CWB-MSSANet remains competitive and relatively similar, SFBS-FFGNET and DIEFEN excel in specific dimensions, securing the highest Pr and Re values, respectively. Notably, the proposed FDS-BINet surpasses all competing methods in OA, Kappa, and F1-score, underscoring its exceptional comprehensive detection capability and categorical consistency. The qualitative visualizations in Fig 7 further corroborate these findings. CVA and PCA-KM show the most pronounced deviations from the Ground Truth (GT), with the former plagued by excessive false positives (erroneous white areas) and the latter by significant missed detections (abnormal black areas). While other deep learning methods align more closely with the GT from a global perspective, a granular inspection reveals that FDS-BINet provides the most precise boundary delineation and is most congruent with the reference map.

2) Quantitative and Qualitative Analysis on the River Dataset: According to the results in Table 3, CVA achieves the maximum Re value; however, its meager Pr (0.5444) indicates a high false alarm rate, where numerous stationary pixels are incorrectly identified as changes. PCA-KM records the lowest scores across all five evaluation metrics, reflecting its limited robustness in complex riverine scenes. Among the competitors, CDFormer manifests a well-balanced performance, while CWB-MSSANet secures the top rank in Kappa and Pr. Nevertheless, FDS-BINet maintains its dominance by achieving the highest OA and F1 values, validating its superior overall efficacy. As illustrated in Fig 8, the CVA detection map is characterized by conspicuous false alarm noise. PCA-KM not only exhibits significant false positives but also fails to reconstruct the semantic structure in the upper-right region compared to the GT. Similarly, the maps produced by 2D-CNN and BCNNs are hindered by prominent erroneous white regions. In contrast, while the remaining advanced methods yield visually similar results to the GT, FDS-BINet demonstrates a superior ability to resolve fine-grained textural details and suppress background interference.

3) Quantitative and Qualitative Analysis on the Hermiston Dataset: The experimental data summarized in Table 4 reveal distinct performance patterns for the Hermiston dataset. Although CVA achieves the highest Pr value, its OA (0.9392) and Re (0.5317) are notably low, resulting in the least effective comprehensive performance among all tested methods. Similarly, PCA-KM reaches a high Pr of 0.9671, yet its limited Re (0.6481) suppresses its Kappa and F1 scores. Among the deep learning models, BCNNs, SSA-SiamNet, and CDFormer exhibit comparable efficacy, with BCNNs attaining the peak Re value. While CWB-MSSANet, SFBS-FFGNET, and DIEFEN show steady results, they do not lead in any specific metric. Notably, the proposed FDS-BINet maintains a slight lead in OA, while its Kappa and F1 scores significantly outperform all baseline methods, demonstrating the highest level of detection consistency. The qualitative results presented in Fig 9 align with the quantitative metrics. Visual inspection reveals substantial omissions (missing white regions) in the classification maps generated by CVA and PCA-KM. While the other deep learning frameworks significantly reduce these errors, the FDS-BINet binary maps demonstrate the highest fidelity to the ground truth. By effectively capturing fine-grained details, FDS-BINet establishes a clear superiority over the existing state-of-the-art comparison methods.

### 3.4 Computational cost analysis

To further assess the practical viability of the proposed framework, we conducted a comparative analysis of computational costs involving five representative deep learning methodologies, as summarized in Table 5. Taking the River dataset as a primary example, FDS-BINet exhibits a compelling advantage in balancing training overhead, inference speed, and model complexity. Specifically, FDS-BINet requires a training duration of 260.45 seconds, which is substantially more efficient than the 782.79 seconds required by SFBS-FFGNET. Its testing phase is equally optimized, with an execution time of only 13.25 seconds, outperforming both 2D-CNN and CWB-MSSANet. Furthermore, FDS-BINet maintains a relatively compact architectural footprint with 292.36k parameters. While this is marginally higher than SSA-SiamNet, it represents a dramatic reduction in complexity compared to the 48.99M parameters of SFBS-FFGNET.

**Table 5:** Computational costs of different CD methods on three dual-temporal HSIS datasets.

Dataset	Method	Training Time (s)	Testing Time (s)	Parameters
River	2D-CNN	210.59s	25.33s	372.88k
	SSA-SiamNet	355.14s	30.87s	107.12k
	CBW-MSSANet	380.69s	49.31s	85.69k
	SFBS-FFGNET	782.79s	11.75s	48.99M
	FBS-BINet	260.45s	13.25s	292.36k
Farmland	2D-CNN	217.65s	15.42s	363.59k
	SSA-SiamNet	325.21	20.11s	97.83k
	CBW-MSSANet	350.20s	24.69s	73.31k
	SFBS-FFGNET	892.18s	20.12s	29.53M
	FBS-BINet	253.88s	10.72s	308.46K
Hermiston	2D-CNN	172.56s	17.51s	382.39k
	SSA-SiamNet	385.42s	22.59s	116.62k
	CBW-MSSANet	370.12s	35.47s	98.36k
	SFBS-FFGNET	865.78s	14.72s	38.48M
	FBS-BINet	233.71s	11.29	312.56k

In conclusion, FDS-BINet demonstrates consistently high computational efficiency across all three benchmark HSI datasets. Its ability to maintain rapid training and testing cycles while preserving superior detection accuracy renders it an exceptionally effective solution for HSI change detection tasks where both performance and resource efficiency are paramount.

### 3.5 Analysis of ablation experiments

The proposed FDS-BINet integrates two pivotal components: the FDS module and the subpixel branch. To quantitatively assess their individual contributions, we designed three ablation models—Siam-DisNet, Siam-BINet, and Siam-FDSNet—for a comparative evaluation across three benchmark datasets.

The Siam-DisNet serves as the baseline where both the FDS module and subpixel branch are removed, and the FDS is replaced by a conventional distance-based feature transformation. As shown in Table 6, Siam-DisNet consistently exhibits the lowest performance across all five metrics. On the Farmland dataset, it significantly lags behind Siam-FDSNet and Siam-BINet, with only the Recall (Re) value showing relative proximity. On the River dataset, despite a narrow gap in OA, its other metrics are substantially lower, indicating poor detection consistency. This phenomenon is attributed to the dataset’s class imbalance; a high OA can still be achieved if the majority unchanged pixels are correctly identified, even with subpar Precision (Pr) and Re. For the Hermiston dataset, while Siam-DisNet exceeds 0.9 in all metrics, its detection consistency remains the weakest. These results collectively demonstrate that the FDS module is essential for effectively integrating Siamese differential features.

**Table 6:** Ablation experiments on three dual-temporal HSIS datasets.

Dataset	Method	OA	Kappa	Pr	Re	F1
River	Siam-DisNet	0.9629	0.7444	0.7243	0.8093	0.7644
	Siam-BINet	0.9711	0.7632	0.7266	0.8216	0.8155
	Siam-FDSNet	0.9679	0.7890	0.7287	0.9023	0.8062
	FDS-BINet	**0.9774**	0.8446	0.8048	0.9161	**0.8568**
Farmland	Siam-DisNet	0.9692	0.9257	0.9321	0.9632	0.9474
	Siam-BINet	0.9705	0.9342	0.9522	0.9675	0.9548
	Siam-FDSNet	0.9773	0.9447	0.9572	0.9642	0.9607
	FDS-BINet	**0.9831**	**0.9588**	0.9702	0.9711	**0.9706**
Hermiston	Siam-DisNet	0.9861	0.9354	0.9037	0.9865	0.9433
	Siam-BINet	0.9876	0.9410	0.9127	0.9880	0.9455
	Siam-FDSNet	0.9879	0.9435	0.9144	0.9894	0.9504
	FDS-BINet	**0.9895**	**0.9510**	0.9252	0.9909	**0.9569**

To isolate the FDS module’s impact while retaining subpixel information, Siam-BINet utilizes conventional distance calculations instead of the FDS module. In the Farmland dataset, while Siam-BINet outperforms Siam-DisNet (particularly in F1-score), it remains inferior to the full FDS-BINet, especially in Re, F1, and Kappa. On other datasets, FDS-BINet consistently achieves superior results, although the performance gap between it and Siam-BINet is more refined than the gap between Siam-BINet and Siam-DisNet. This suggests that while subpixel information provides a strong foundation, the FDS module is the critical driver for achieving state-of-the-art precision.

Siam-FDSNet excludes the subpixel branch to evaluate the necessity of fine-grained spatial cues. Experimental results in Table 6 for the Farmland dataset reveal that all metrics for Siam-FDSNet are significantly lower than those of FDS-BINet. In the River dataset, although OA and Re remain relatively stable, the other three metrics fall considerably behind, reflecting an inability to resolve complex transitions. Similarly, on the Hermiston dataset, Siam-FDSNet displays unbalanced performance, with Kappa, Pr, and F1 lagging by a substantial margin. These comprehensive comparisons confirm that the subpixel branch provides indispensable supplementary information that enhances detection robustness and detail resolution.

### 3.6 Parameter sensitivity analysis

Unless otherwise specified, the spatial patch size, subpixel patch size, interpolation rate, and number of convolutional filters are fixed as (9×9), (9×9), 2, and 32, respectively. The configuration of hyperparameters plays a pivotal role in determining the final performance of the model. To identify the optimal parameter combination, we conducted extensive sensitivity experiments focusing on the number of kernels, batch size, and training set ratio.

**Fig 10:**
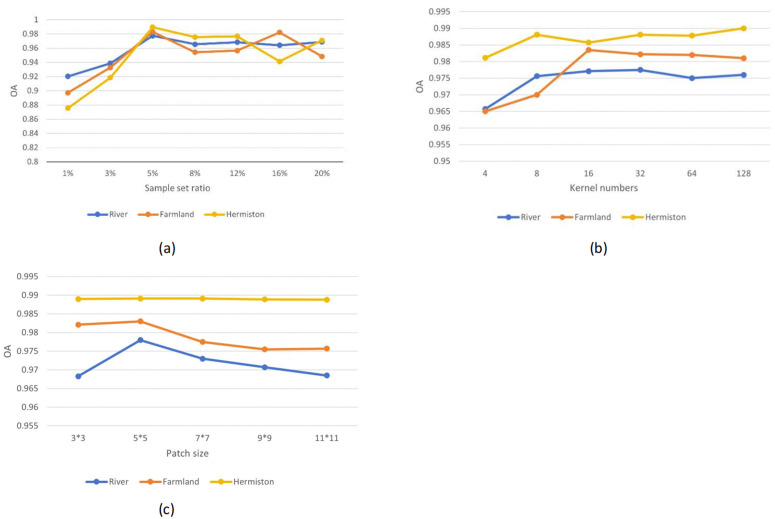
OA curves of different parameter changes of FDS-BINet on three datasets.

For kernel numbers, we evaluated six different kernel counts {4, 8, 16, 32, 64, 128}. As depicted by the OA curves in Fig 10(a), all three datasets exhibit an upward performance trend as the kernel count increases from 4 to 16. Beyond this point, the accuracy for the Hermiston dataset stabilizes, while Farmland and River continue to show improvement. Peak OA is universally achieved at a kernel count of 32. Since higher values lead to increased computational load without yielding significant gains in accuracy, we selected 32 as the optimal kernel number.

For batch size, we set five different values {16, 32, 64, 128, 256}. According to Fig 10(b), the OA curves for the River and Hermiston datasets decline progressively as the batch size increases. Although the Farmland dataset shows slight fluctuations, it nonetheless reaches its maximum OA at a batch size of 16. Consequently, the batch size was fixed at 16 for all subsequent experiments.

For training set ratio, We conducted experiments with varying training set ratios (1%, 3%, 5%, and 10%) on the dataset. The results in Fig 10(c) indicate that FDS-BINet reaches optimal precision at a 5% ratio, after which the performance plateaus with only marginal fluctuations. This analysis confirms that the method is highly efficient even with limited labeled data and demonstrates strong robustness across varying training scales.

## 4 Conclusion

In this paper, we propose FDS-BINet, a novel framework for hyperspectral image (HSI) change detection (CD). The primary innovation lies in the development of the Feature Difference Suppression (FDS) module, which optimizes the extraction of discriminative differential features within a Siamese network architecture. Simultaneously, we introduce a subpixel branch powered by the Bicubic Interpolation (BI) algorithm to incorporate fine-grained spatial cues often overlooked in conventional methods. To further refine feature representation, we integrate Channel Weighting (CW) and Spatial Weighting (SW) modules for dynamic information redistribution, followed by a dedicated Feature Extraction (FE) module. The efficacy of FDS-BINet was rigorously validated against two algebraic-based and seven representative deep learning-based methodologies. Experimental results across multiple benchmark datasets demonstrate that FDS-BINet achieves state-of-the-art detection performance. Furthermore, specialized ablation studies confirm that both the FDS module and subpixel spatial information independently and significantly contribute to the enhancement of CD accuracy. Finally, through extensive parameter sensitivity analysis, we identified the optimal hyperparameter configuration, ensuring the model’s robustness and efficiency.

## Supporting information

S1 Data Dataset. (XLSX)
